# Colorimetric Detection of Indoxyl Sulfate With Sodium Hypochlorite Pentahydrate Analyzed by Spectrophotometry and ESI‐MS

**DOI:** 10.1155/ianc/8276950

**Published:** 2026-11-02

**Authors:** Taeko Nakagawa, Kazuaki Ohara, Kentaro Yamaguchi, Yasunori Tokuhara

**Affiliations:** ^1^ Department of Medical Technology Graduate School of Health Sciences Kagawa Prefectural University of Health Sciences Takamatsu Kagawa Japan kagawa.jp; ^2^ Faculty of Pharmaceutical Sciences at Kagawa Campus Tokushima Bunri University Takamatsu Kagawa Japan bunri-u.ac.jp

**Keywords:** absorbance, ESI-MS, indican, indigo, Jaffé method, Obermayer method, sodium hypochlorite pentahydrate (NaOCl·5H_2_O)

## Abstract

Indoxyl sulfate (indican) is a tryptophan‐derived metabolite generated by intestinal bacteria and subsequently excreted in urine. Urinary indican is considered an indicator of gut microbial imbalance and small‐intestinal dysfunction because its production closely reflects the extent of microbial activity. In this study, we improved the classical indican color reaction—originally based on the Obermayer and Jaffé methods—by developing a simple colorimetric method using sodium hypochlorite pentahydrate (NaOCl·5H_2_O) as a novel oxidizing agent that is characterized by its high stability, strong reactivity, and ease of handling. By using this new oxidizing agent, the overall detection procedure becomes simpler to perform than the classical methods. The addition of hydrochloric acid followed by NaOCl·5H_2_O produced a blue pigment with an absorbance peak at approximately 600 nm, which was identified as indigo by electrospray ionization mass spectrometry. The method exhibited a visual detection limit of 6 mg/dL, comparable to that of the Obermayer and Jaffé methods. In the proposed method, a linear response between 6 and 12 mg/dL (*R* ^2^ = 0.9369) was obtained, and the recovery in spiked human urine samples was 69.3%; however, these results indicate that further improvement is required for reliable quantification of elevated indican levels. Moreover, the chromogenic reaction was susceptible to interference from ascorbic acid although partial suppression was achieved using ascorbate oxidase. Overall, this work provides a useful analytical approach for detecting elevated urinary indican levels in clinical and research settings, serving as an alternative to classical methods that rely on ferric chloride and calcium hypochlorite.

## 1. Introduction

Indoxyl sulfate (indican) is a tryptophan‐derived metabolite produced through bacterial fermentation in the intestinal tract, and following absorption from the intestine, it is converted in the liver and subsequently excreted in the urine [1–4]. As the production of indican depends on intestinal microbial activity, urinary indican has long been regarded as a potential indicator of gut microbial imbalance and small‐intestinal dysfunction, thereby attracting considerable attention in clinical diagnostics and intestinal research [5–7].

Indican can be quantified based on several analytical techniques, including mass spectrometry, high‐performance liquid chromatography (HPLC), and colorimetry [8–12]. One of the classical colorimetric methods involves hydrolyzing indican with hydrochloric acid (HCl) to generate indoxyl and subsequently oxidizing it to produce indigo (Figure 1); ferric chloride is used as the oxidizing agent in the Obermayer method [13, 14], whereas calcium hypochlorite is used in the Jaffé method [15, 16].

**FIGURE 1:**
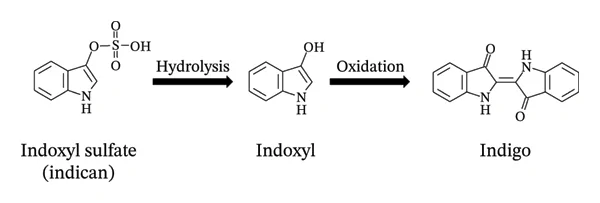
The reaction process leading from indoxyl sulfate (indican) to the formation of indigo.

Sodium hypochlorite pentahydrate (NaOCl·5H_2_O) is an oxidant that is commonly supplied as a high‐purity, crystalline material [17]. In previous experiments, we established spectrophotometric methods for detecting amino acid metabolites and structurally related compounds using NaOCl·5H_2_O, demonstrating its utility as an oxidizing agent and highlighting its high stability, high reactivity, and ease of handling [18–20]. Accordingly, we aimed to improve the indican color reaction by employing NaOCl·5H_2_O as the oxidizing agent.

In the present study, we developed a simple approach using NaOCl·5H_2_O to detect indican and examined the characteristics of the absorption spectra by spectrophotometry. We also performed the first comparative analysis of the Obermayer and Jaffé methods applying electrospray ionization mass spectrometry (ESI‐MS), verifying the formation of the indigo pigment among all three methods. Furthermore, we investigated the interference from ascorbic acid (AA), an antioxidant commonly found in physiological samples. Ultimately, this method may serve as a new approach for indican detection.

## 2. Materials and Methods

### 2.1. Reagents and Equipment

Indoxyl sulfate (indican) and sodium hypochlorite pentahydrate (NaOCl·5H_2_O) were purchased from Nacalai Tesque (Kyoto, Japan). Indigo was purchased from Tokyo Chemical Industry (Tokyo, Japan). Ferric chloride (FeCl_3_), calcium hypochlorite (Ca(OCl)_2_), 10% HCl, chloroform, methanol, 0.1 mol/L phosphate buffer solution (pH 7.0), AA, and ascorbate oxidase (AO) were purchased from FUJIFILM Wako Pure Chemical Corporation (Osaka, Japan). Urine from a single donor (Cat. No. 991‐03‐S) was purchased from Medix Biochemica (Cambridge, MA, USA). Absorption spectra were measured using a double‐beam U‐2900 spectrophotometer (Hitachi High‐Tech, Tokyo, Japan) and microcells with a 10‐mm path length. ESI‐MS was performed using a magnetic resonance mass spectrometer (scimaX, Bruker Daltonics GmbH & Co. KG, Bremen, Germany).

### 2.2. Preparation of the Samples and Experimental Reagents

A stock 100 mg/dL indican solution was prepared by dissolution in purified water; samples were obtained by diluting the solution to 30, 28, 26, 24, 22, 20, 18, 16, 14, 12, 10, 9, 8, 7, 6, 5, 4, 3, 2, and 1 mg/dL with purified water. A 1000 mg/dL FeCl_3_ solution was prepared by dissolution in 10% HCl. Solutions of Ca(OCl)_2_ (1000 mg/dL) and NaOCl·5H_2_O (1000 mg/dL) were prepared by dissolving each compound in purified water.

### 2.3. Color Change Observation and the Spectrophotometric Procedure

For the Obermayer method, 300 μL of 1000 mg/dL FeCl_3_ solution was added to 300 μL of each sample and mixed. For the Jaffé method, 300 μL of 10% HCl was added to 300 μL of each sample and mixed, followed by 3 μL of 1000 mg/dL Ca(OCl)_2_ with mixing. For the method presented herein, 300 μL of 10% HCl was added to 300 μL of each sample and mixed, followed by 3 μL of 1000 mg/dL NaOCl·5H_2_O with mixing. Each method was incubated at room temperature for 10 min, and the blue pigment was extracted with 600 μL of chloroform. The color change of the substances extracted into the chloroform layer was observed using the U‐2900 spectrophotometer, with a 1‐nm bandwidth and a scan speed of 100 nm/min.

To evaluate the linearity range, 300 μL of an aqueous indican solution (1–30 mg/dL) was mixed with 300 μL of 10% HCl, followed by the addition of 3 μL of 1000 mg/dL NaOCl·5H_2_O with mixing. After incubation at room temperature for 10 min, the absorbance of the chloroform‐extracted sample was measured at 600 nm. The standard deviation (SD) of the blank was obtained from seven replicate measurements of the reagent blank (purified water). The limit of detection (LOD) and limit of quantification (LOQ) were calculated according to ICH Q2 guidelines [21] using the following equations: LOD = 3.3 × SD of the blank/slope and LOQ = 10 × SD of the blank/slope. The slope was obtained from the calibration curve constructed using standards between 6 and 12 mg/dL.

Samples were prepared for the spiking recovery test of indican (7 mg/dL) by adding one volume of a 70 mg/dL aqueous indican solution to nine volumes of donor urine. Each sample was then mixed with 300 μL of 10% HCl and 3 μL of 1000 mg/dL NaOCl·5H_2_O. After incubation at room temperature for 10 min, the absorbance at 600 nm was measured, and the percent recovery was calculated.

To examine the effects of an antioxidant, 300 μL of 10% HCl was added to 300 μL of 50 mg/dL indican containing AA (100, 200, 300, 400, or 500 mg/dL), followed by 3 μL of 1000 mg/dL NaOCl·5H_2_O with mixing. Additionally, to investigate the effect of AO, a 200 U/mL AO solution prepared by dissolving AO in phosphate buffer (pH 7.0) was added to 300 μL of a 50 mg/dL indican solution containing AA (100, 200, 300, 400, or 500 mg/dL), yielding a final AO concentration of 50 U/mL. To this sample, 300 μL of 10% HCl was added, followed by 3 μL of 1000 mg/dL NaOCl·5H_2_O with mixing. The mixed samples were then incubated at room temperature for 10 min, and the blue pigment was extracted with 600 μL of chloroform. The absorbance change of the substances extracted into the chloroform layer was observed using the U‐2900 spectrophotometer.

### 2.4. ESI‐MS

A solution prepared by dissolving 1 mg of indigo in 10 mL of chloroform was mixed with methanol at a ratio of 4:1 and analyzed by Fourier transform ion cyclotron resonance mass spectrometry using the scimaX. Furthermore, 100 mg/dL aqueous indican solutions that turned blue using the Obermayer method, the Jaffé method, and the proposed method, followed by chloroform extraction, were each mixed with methanol at a ratio of 4:1 and subsequently analyzed in the same manner. Mass spectra were calibrated using external calibration with a tuning‐mix (Agilent, Santa Clara, CA, USA). The following instrument parameters were used: the sample flow rate was 3 μL/min, the dry gas plate temperature was 250°C, the rate of N_2_ drying gas was 4.0 L/min, the rate of N_2_ nebulizing gas was 1.0 bar, and the capillary voltage was 4.5 kV for the positive ion detection mode.

## 3. Results and Discussion

### 3.1. Comparison of the Obermayer, Jaffé, and Proposed Methods

FeCl_3_ is used as the oxidizing agent in the Obermayer method, whereas Ca(OCl)_2_ is used in the Jaffé method [13–15]. In this study, however, we investigated a new method for producing indigo from indican by using NaOCl·5H_2_O as a novel oxidizing agent.

We first compared the color changes and absorbance of the products obtained using the Obermayer, Jaffé, and proposed methods. The colorless aqueous solution composed of indican (100 mg/dL) turned to a comparable blue color for all three methods (Figure 2(a)). We then measured the absorption spectra (500–700 nm) of the samples, which each showed an absorbance peak near 600 nm (Figure 2(b)). Thus, NaOCl·5H_2_O enabled a color reaction comparable to that of the Obermayer and Jaffé methods. Moreover, considering their similar absorption curves, the proposed method is equally useful as the previous methods for detecting indican. To fully assess the practical applicability of the proposed method, the stability and reproducibility of NaOCl·5H_2_O must be considered, in addition to its analytical performance.

**FIGURE 2:**
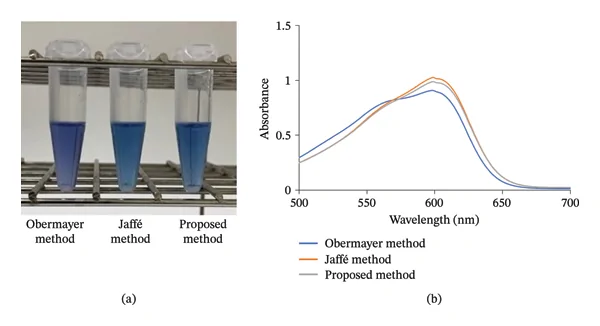
(a) Color changes and (b) absorption spectra of 100 mg/dL aqueous indican solutions reacted using the Obermayer method, the Jaffé method, and the proposed method.

Stability is essential for routine use as an analytical oxidant. Previous work has demonstrated that NaOCl·5H_2_O crystals exhibit markedly higher storage stability than conventional aqueous NaOCl solutions. Kirihara et al. reported that NaOCl·5H_2_O retains approximately 99% of its available chlorine content after 360 days at 7°C, whereas a 13.6% aqueous NaOCl solution stored under the same conditions decreases to 83% of its initial concentration [17]. The crystalline form contains only 0.04–0.08% NaOH and allows accurate mass‐based preparation of solutions, minimizing batch‐to‐batch variability. In our study, all NaOCl·5H_2_O solutions prepared from crystalline material produced consistent colorimetric responses and comparable absorbance spectra, indicating that the oxidizing performance is highly reproducible across different preparations. These characteristics support the suitability of NaOCl·5H_2_O as a stable and reliable oxidant for routine indican analysis.

### 3.2. ESI‐MS Analysis

Next, we employed high resolution (resolution *R* ∼200,000) ESI‐MS to determine whether indigo, a blue pigment, was formed from indican using the Obermayer, Jaffé, and proposed methods. Authentic indigo (C_16_H_10_N_2_O_2_) has a protonated molecular ion peak at m/z 263.0811, consistent with the calculated exact mass for C_16_H_11_N_2_O_2_ ([M+H]^+^, 263.0810) (Figure 3(a)). The Obermayer sample exhibited an ion peak at m/z 263.0813 (Figure 3(b)), and the Jaffé sample showed the same protonated molecular ion peak at m/z 263.0814 (Figure 3(c)). Similar protonated molecular ion peaks with comparable ion intensities were observed for the sample obtained using the proposed method (Figure 3(d)). These ESI‐MS results indicate that indigo with the expected elemental composition was formed using the Obermayer, Jaffé, and proposed methods. Additionally, all blue‐colored indican solutions contained indigo, confirming that the same oxidative reaction occurred. The findings suggest that the proposed method using NaOCl·5H_2_O is an effective alternative to the Obermayer and Jaffé methods.

**FIGURE 3:**
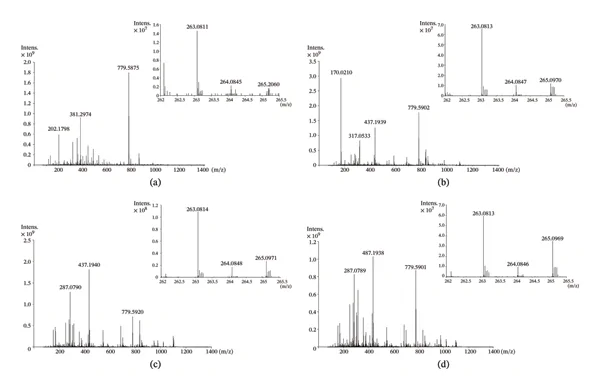
(a) High resolution (resolution *R* ∼200,000) ESI‐MS spectra of 10 mg/dL indigo, and 100 mg/dL aqueous indican solutions that turned blue using (b) the Obermayer method, (c) the Jaffé method, and (d) the proposed method. Expanded views of the m/z 262.0–265.5 ranges are shown in the insets, along with the peak assignments.

Recent studies have highlighted the diverse reactivity and mechanistic behavior of hypochlorite species in organic and biological systems [22], providing a broader context for the oxidative processes observed in our assay. Several reports have demonstrated that hypochlorite can participate in selective oxidation, electrophilic chlorination, and radical‐mediated pathways depending on the molecular environment and reaction conditions [23]. For example, recent mechanistic investigations have clarified hypochlorite‐induced oxidative transformations of aromatic and heterocyclic compounds, as well as their interactions with biomolecules [24]. These findings suggest that NaOCl·5H_2_O can efficiently oxidize indoxyl, ultimately yielding indigo, as observed in our study.

Moreover, we analyzed the reaction details of the sample prior to the addition of NaOCl·5H_2_O (i.e., the indican solution after the addition of 10% HCl), employing ESI‐MS. In the positive ion mode, a peak corresponding to indoxyl (C_8_H_7_NO), [M+H]^+^ at m/z 134.0601, was detected, as shown in Supporting Figure S1. This result indicates that acid, such as HCl, is required to hydrolyze indican to indoxyl (Figure 1) and that the resulting indoxyl is subsequently oxidized by NaOCl·5H_2_O to produce indigo (Figure 3(d)).

### 3.3. Color Change and Absorption Spectra of the Samples With Different Indican Concentrations

We then compared the color changes of indican samples at various concentrations using the Obermayer method, the Jaffé method, and the proposed method. Aqueous solutions of indican at 5, 6, 7, 8, 9, 10, and 20 mg/dL were treated using these methods, and the colors of the chloroform extracts were evaluated. In the Obermayer method, a blue‐toned color change was visually observed at indican concentrations of 6–20 mg/dL, and no distinct color change was detected at lower concentrations (Figure 4(a)). In the Jaffé method, blue coloration was confirmed at concentrations of 8–20 mg/dL (Figure 4(a)). In the proposed method, color changes to blue were visually confirmed for indican concentrations of 6–20 mg/dL, and no color change was observed at 5 mg/dL (Figure 4(a)).

**FIGURE 4:**
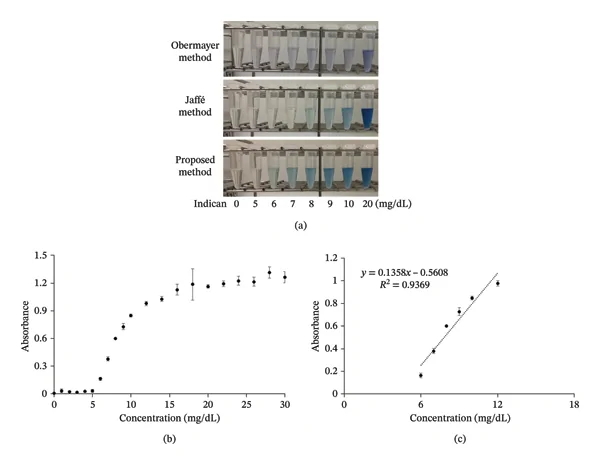
(a) Color changes of aqueous indican solutions with different concentrations (0, 5, 6, 7, 8, 9, 10, and 20 mg/dL) reacted using the Obermayer method, the Jaffé method, and the proposed method. (b) Absorbance at 600 nm was measured for indican solutions ranging from 1 to 30 mg/dL (1, 2, 3, 4, 5, 6, 7, 8, 9, 10, 12, 14, 16, 18, 20, 22, 24, 26, 28, and 30 mg/dL) after using the proposed method. Results are expressed as the mean ± SD of three experiments. (c) Correlation between indican concentration (6–12 mg/dL) and absorbance (600 nm). Results are expressed as the mean ± SD of three experiments.

According to Cassani et al. [25], the urinary indican concentration in 50 healthy individuals was 2.49 ± 0.46 mg/dL. Urinary indican levels are influenced by the intestinal environment and vary depending on the number of lactobacilli [26]. Aarbakke et al. reported that patients with stagnant loop syndrome excrete more than 100 mg/24 h of urinary indican [27]. Furthermore, urinary indican excretion exceeding 100 mg/24 h has been proposed as a useful indicator for assessing bacterial overgrowth in the upper jejunum [28]. Elevated urinary indican levels have also been reported in constipation and Alzheimer’s disease [25]. Although the visual detection limits of the Obermayer method, the Jaffé method, and the proposed method ranged from 6 to 8 mg/dL (Figure 4(a)), this threshold should be interpreted considering the intended clinical application of the assay. Because healthy individuals typically exhibit urinary indican levels of 2‐3 mg/dL [25], our colorimetric method is not suitable for quantifying subtle physiological variations within the normal range. Instead, it is useful for detecting abnormally elevated indican levels, which are clinically relevant in small‐intestinal bacterial overgrowth, stagnant loop syndrome, constipation, and neurodegenerative disorders, where urinary indican can markedly increase. The proposed NaOCl·5H_2_O‐based assay reliably identifies concentrations exceeding the upper limit of normal (≥ 6 mg/dL), positioning it as a simple screening tool for elevated indican, whereas the detection of mild or early increases may require more sensitive analytical techniques such as HPLC or LC‐MS. To further characterize the analytical performance of the assay beyond visual inspection, we next evaluated the quantitative behavior of the chromogenic reaction using absorbance measurements.

Specifically, we measured the absorbance values at 600 nm for aqueous indican solutions with concentrations of 1, 2, 3, 4, 5, 6, 7, 8, 9, 10, 12, 14, 16, 18, 20, 22, 24, 26, 28, and 30 mg/dL (Figure 4(b)). The absorbance increased with the indican concentration above 6 mg/dL, but the increase became more gradual at concentrations exceeding 12 mg/dL (Figure 4(b)). This saturation‐like behavior suggests that the chromogenic reaction approaches its upper dynamic range at higher concentrations. A linear correlation was observed between 6 and 12 mg/dL (*R* ^2^ = 0.9369), indicating that this concentration range represents the most reliable quantitative window for the proposed assay (Figure 4(c)). Based on the regression line (*y* = 0.1358*x*−0.5608), the LOD and LOQ were calculated to be 2.85 and 8.65 mg/dL, respectively (SD of the blank: 0.12 mg/dL). Although these values are comparable to or lower than the visual detection threshold, the practical quantifiable range is constrained by the linearity of the colorimetric response. Therefore, while the theoretical sensitivity of the assay is high, quantitative interpretation should be limited to concentrations within the linear range.

Furthermore, recovery experiments demonstrated that the proposed method exhibits acceptable analytical performance in a biological matrix, namely human urine. The recovery ratio was 69.3% (Table 1) with a relative SD of 4.1% (*n* = 3, 6.0 ± 0.2 mg/dL), indicating that the assay provides good precision but somewhat low accuracy, thus requiring further improvement for reliable screening of elevated urinary indican levels in real‐world clinical specimens. Moreover, further optimization of the chromogenic reaction conditions is necessary to improve the analytical performance. Refining the reaction parameters may help expand the linear dynamic range beyond 6–12 mg/dL, enhance the robustness of the calibration curve, and increase the recovery ratio in biological matrices. Such improvements would contribute to establishing a more reliable and versatile colorimetric method for urinary indican analysis.

**TABLE 1:** Spectrophotometric determination of indican in the human urine sample (*n* = 3).

Sample	Spiked concentration (mg/dL)	Measured concentration (mg/dL, mean ± SD)	RSD (%)	Recovery (%)
Urine	0	1.3 ± 0.1	5.4	—
	7	6.0 ± 0.2	4.1	69.3

### 3.4. Effect of AA

The potential interference from AA, an antioxidant commonly found in urine samples, was investigated because its reducing properties may compete with NaOCl·5H_2_O‐mediated oxidation. By adding various concentrations of AA (100, 200, 300, 400, and 500 mg/dL) to aqueous indican solutions and observing the color changes induced using the proposed method, we found that the blue coloration became weaker as the AA concentration increased (Figure 5(a)), and the absorbance at 600 nm decreased correspondingly (Figure 5(b)). We further examined whether the influence of AA could be suppressed by using AO. When AO was added to each sample at a final concentration of 50 U/mL, the decrease in color development caused by AA at 100 mg/dL was successfully inhibited (Figure 5(a)), and the absorbance at 600 nm also increased (Figure 5(b)). However, when the AA concentration exceeded 200 mg/dL, the influence of AA could no longer be suppressed, and no substantial change in absorbance at 600 nm was observed (Figure 5(b)).

**FIGURE 5:**
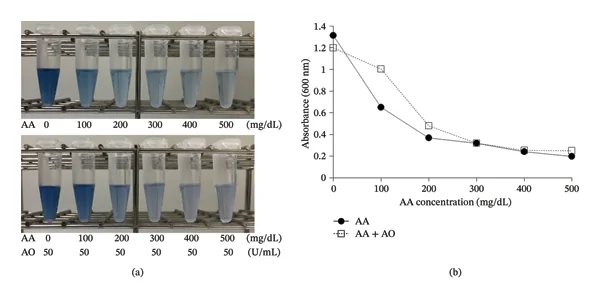
(a) Color changes and (b) absorbance at 600 nm for 50 mg/dL aqueous indican solutions containing different concentrations of ascorbic acid (AA) (0, 100, 200, 300, 400, or 500 mg/dL), with or without 50 U/mL ascorbate oxidase (AO).

AA, also known as vitamin C, is a water‐soluble nutrient found in foods and beverages; therefore, excessive intake is excreted in the urine [29–31]. Consequently, urine containing high levels of AA can cause false‐negative results for dipstick test parameters such as blood, glucose, bilirubin, and nitrite [32–34]. Considering that the chromogenic response of the proposed method was diminished by AA, high urinary concentrations of AA may lead to false‐negative results. Although the influence of AA can be partially suppressed by using AO (Figure 5), this inhibitory effect is limited; when the AA concentration is sufficiently high, AO can no longer counteract the interference, and this limitation should be considered when interpreting the results.

## 4. Conclusions

We examined the application of NaOCl·5H_2_O as a novel oxidizing agent in colorimetric indican detection. Spectrophotometric and ESI‐MS analyses revealed that, similar to the Obermayer and Jaffé methods, the proposed method detects indican through the oxidative formation of indigo. These findings indicate that NaOCl·5H_2_O can serve as an alternative oxidizing agent for urinary indican tests.

## Author Contributions

Taeko Nakagawa: conceptualization, investigation, data acquisition, data analysis, writing–original draft and review and editing, and final approval. Kazuaki Ohara: investigation, data acquisition, data analysis, writing–review and editing, and final approval. Kentaro Yamaguchi: supervision, data analysis, writing–review and editing, and final approval. Yasunori Tokuhara: supervision, project administration, funding acquisition, investigation, data acquisition, data analysis, writing–original draft and review and editing, and final approval.

## Funding

This work was supported by JSPS KAKENHI Grant number JP24K10602.

## Disclosure

All authors have read and agreed to the published version of the manuscript.

## Conflicts of Interest

The authors declare no conflicts of interest.

## Supporting Information

Additional supporting information can be found online in the Supporting Information section.

## Supporting information


**Supporting Information** Supporting Figure S1 shows the ESI‐MS spectra of an aqueous indican solution after the addition of HCl.

## Data Availability

The data that support the findings of this study are available from the corresponding author upon reasonable request.
