# Supplementary material for: Colorimetric Detection of Indoxyl Sulfate With Sodium Hypochlorite Pentahydrate Analyzed by Spectrophotometry and ESI‐MS
Source: Int J Anal Chem. 2026 Nov 2;2026:8276950. doi: 10.1155/ianc/8276950 (PMC13725846; doi:10.1155/ianc/8276950)
Supplement: Supplementary file 1 [file IANC-2026-8276950-s001.docx]

**Colorimetric Detection of Indoxyl Sulfate with Sodium Hypochlorite Pentahydrate Analyzed by Spectrophotometry and ESI‑MS**

Taeko Nakagawa^1^, Kazuaki Ohara^2^, Kentaro Yamaguchi^2^, Yasunori Tokuhara^1^

^1^ Department of Medical Technology, Graduate School of Health Sciences, Kagawa Prefectural University of Health Sciences

^2^ Faculty of Pharmaceutical Sciences at Kagawa Campus, Tokushima Bunri University, Kagawa, Japan

**Correspondence**

Yasunori Tokuhara, Department of Medical Technology, Graduate School of Health Sciences, Kagawa Prefectural University of Health Sciences, 281-1 Hara, Mure-cho, Takamatsu, Kagawa 761-0123, Japan

E-mail: tokuhara-y@kagawa-puhs.ac.jp

Figure S1: ESI-MS spectra of 300 µL of 100 mg/dL aqueous indican solutions after the addition of 300 µL of 10% HCl. Expanded views of the *m/z* 132.0–137.0 ranges are shown in the insets, along with the peak assignments.
